# The study and date analysis of interictal electroencephalogram in epileptic patients

**DOI:** 10.1186/1471-2202-12-S1-P166

**Published:** 2011-07-18

**Authors:** Irma Khachidze

**Affiliations:** 1 Ivane Beritashvili Institute of Physiology. Life Sciences Research Center. Tbilisi, Georgia

## 

The modern anticonvulsants can imitate normalization of clinical futures in epileptic children and change the typical epileptiform pattern of electroencephalogram (EEG). For this reason the correct evaluation of EEG in interictal period gain a special significance for adequate treatment strategy. The aim of this study was to elaborate the criteria for evolution of interictal EEG patterns using a computer EEG approach. EEG permits to study the correlation between clinical and neurophysiological outcomes making this technique useful not only for monitoring the course of the treatment but also for predicting the degree of tolerance and evaluating probable side effects, which is very important for revealing the possible early predictors of benefit/adverse effects of the treatment and for optimizing the anti-epileptic therapy. This study was aimed at investigating the alteration of different EEG characteristics in epilepsy contingent upon the treatment.

The quantitative analysis of power spectrum of all frequencies of EEG potentials was analyzed. EEG signals were digitally recorded using a set of 19 scalp electrodes according to the International 10–20 system and ANCEPHALAN 131-03, professional version “MEDICOM”.

For the statistical evaluation, the EEG phenomena were calculated within 6 frequency bands. The following quantitative characteristics of the EEGs were analyzed: 1) the pattern of native EEG with monopolar, bipolar and common average reference montage recordings for the evaluation of the specificity of the background activity, 2) absolute values of the power spectra, 3) mapping of the EEG spectral characteristics (topography). Qualitative assessment of the following EEG characteristics was performed: 1) interictal epileptiform abnormalities–with this the spike density and the number of paroxysmal discharges, 2) characteristics of alpha activity, 3) characteristics of beta activity. 137 patients with different forms of epilepsy, aged 3 to 12 years were examined. During the observation all patients received different types of anticonvulsants.

## Results

Quantitative Spectral analysis of interictal EEG reveals that in the total EEG spectrum the most powerful are the oscillations of 3-8 Hz with prevalent amplitude of 60-120 μv^2^. The essential prognostic value has the morphology of the theta-waves and its distribution upon the convex cortical surface: the presence of monomorphic mid and high amplitude theta-waves especially in the temporoparietal regions allows us to expect the re-occurrence of seizures should the anticonvulsants be canceled.

## Conclusions

The presence of monomorphic mid and high amplitude theta-waves of temporo-parietal localization in the interictal EEG of the children treated by anticonvulsants is a negative finding despite of the normalization of a patient clinical status. The computer EEG analysis of interictal EEG pattern in epileptic children treated with anticonvulsants allows to correctly evaluate the treatment strategy. The value of EEG date as predicting seizure exacerbation in children with new onset epilepsy is very important. In this way, any antiepileptic therapy should be performed with maximal caution and under regular EEG-control. Because, worsening of EEG-characteristics, in some cases, precedes the onset of clinical signs of exacerbation of the patient’s state. Careful follow-up EEG, including repeated EEG recordings and date analysis will be useful to identify changes predictive of seizures aggravation after initiation of treatment.

